# Recurrence of arteriovenous malformations of the brain after complete surgical resection. Kuopio University Hospital experience and systematic review of the literature

**DOI:** 10.1007/s10143-023-02001-8

**Published:** 2023-04-29

**Authors:** Patrik Järvelin, Henri Pekonen, Timo Koivisto, Juhana Frösen

**Affiliations:** 1 Hemorrhagic Brain Pathology Research Group, Kuopio University Hospital and Tampere University, Tampere, Finland; 2 Dept of Neurosurgery, Kuopio University Hospital, Kuopio, Finland; 3 Dept of Neurosurgery, Tampere University Hospital, Tampere, Finland

**Keywords:** Arteriovenous malformation, bAVM, Recurrence, Hemorrhage, Pediatric

## Abstract

Treatment for arteriovenous malformations of the brain (bAVMs) aims to achieve complete removal or occlusion of the lesion in order to eradicate the risk of rupture and subsequent morbidity associated with these lesions. Despite initially successful treatment, bAVMs may carry a risk of recurrence especially in younger patients. We studied the rate of recurrence of surgically treated bAVMs at Kuopio University Hospital (KUH) in 1981–2021. The study population was collected retrospectively from KUH databases and presented a cohort of 135 surgically treated bAVMs with complete occlusion of the lesion. We also performed a systematic literature review on this topic. In our series, 6 out of 135 (4.4%) patients with angiographically confirmed removal of the lesion later developed a recurrent bAVM with a median time to diagnosis of recurrence of 7.46 years. In pediatric patients, the rate was 5 out of 17 (29.4%). bAVM recurrence was associated with age (*p* = 0.001) and initial hemorrhagic presentation (*p* = 0.039). Median age of the study population was 37 years (min 0, max 70), and 51/135 (37.8%) of the patients were female. Seventeen (12.6%) of the 135 bAVM patients were considered pediatric (18 years old or younger) at the time of the operation. In the literature review, 79 of 1739 (4.5%) of surgically treated patients later developed a recurrence with a mean delay of 3.1 years until diagnosis of recurrence. Young surgically treated bAVM patients with a hemorrhagic presentation at initial diagnosis are at a relatively high risk of bAVM recurrence. Follow-up imaging should be arranged for these patients in order to prevent rupture from a recurrent bAVM and subsequent morbidity.

## Introduction

Arteriovenous malformations of the brain (bAVMs) are rare lesions with an incidence of approximately 1 per 100,000 person-years in unselected populations [1]. Despite their low incidence, they are the most common cause of intracranial hemorrhage in children [2] and, due to mostly affecting working-aged people, have a larger impact on working years lost due to disease-related morbidity than their prevalence would imply. Surgery, endovascular embolization, and radiosurgery (with either LINAC or gamma knife -based techniques) are the main methods of treatment with which medical professionals tackle these lesions. When successful, surgery eliminates the risk of rupture from the bAVM immediately, while the effect of radiosurgery is delayed and the risk of rupture stays present until occlusion of the lesion, which is usually achieved several years after treatment. bAVMs are highly heterogeneous, with both surgical and radiosurgical risk and success depending greatly on the anatomy of the lesion. Several grading scales have been suggested to assess treatment risk associated with an individual bAVM, with the Spetzler–Martin and Spetzler–Ponce grades [3, 4] being the most commonly used.

While the tendency for bAVMs to grow, especially in pediatric patients, was first brought to attention by Olivecrona et al. [5], bAVM recurrence has been considered a rare phenomenon which has only started to gather more attention within the last 20–30 years. In addition to mostly presenting with patients treated at a young age (pediatric patients), several studies have associated recurrent lesions with initial hemorrhagic presentation [6–8]. The incidence of recurrence creates an additional risk for the patients in question, and accurate data on the prevalence of recurrence is crucial for assessing clinical risk associated with different treatment modalities and for designing follow-up schemes.

## Materials and methods

### Formation of the study cohort from the KUH bAVM registry

KUH bAVM registry was created by a systematic search of the KUH hospital discharge registry for ICD-10 diagnosis codes Q28.0–28.3, complemented by a retrospective review of operating room log books ranging to year 1981. Following this, the medical records and imaging studies of all identified patients were reviewed. Confirmed bAVM cases were entered into the registry, and for them the date of diagnosis, date of treatment, age at first treatment, gender, and neurological symptoms were recorded as described in the patient’s medical records at the time of diagnosis or leading to the diagnosis. Additionally, the given treatments, dates of interventions, and possible dates of ruptures and recurrence as well as postoperative neurological condition were recorded. Post-treatment clinical outcome was assessed according to Glasgow Outcome Scale (GOS), the occurrence of new neurological symptoms, and the patient’s ability to return to work.

For this study, we collected all surgically treated patients from the KUH bAVM registry.

### Treatment and follow-up

In our institution, bAVM patients are managed on a case-by-case basis with treatment recommendations being provided by a multidisciplinary team consisting of neurosurgeons and neuroradiologists. The patients in our registry come from multiple eras with different treatment and follow-up protocols, with the oldest having been initially treated in 1981. Complete removal of the lesion was always confirmed with postoperative DSA. Follow-up imaging was performed if the patient’s neurological condition deteriorated or if the patient presented new symptoms. More recently, long-term follow-up imaging with MRI at approximately 5 and 10 years after treatment has been performed for patients under 40 years of age. We examined our patient follow-up in three different ways. First, we looked at imaging follow-up which covers the period of time from surgical excision of the lesion to recurrence or the latest MRI or DSA imaging performed on the patient. Second, we looked at clinical follow-up which covers the period of time from surgical excision to recurrence or the latest clinical visit or call to either our neurosurgical or neurological clinic. Third, we looked at follow-up based on patient records which covers the period of time from surgical excision of the lesion to the day on which patient records were examined, or the date of death, or the last clinical visit in our hospital if the patient has moved away from the KUH catchment area, or detection of recurrence, whichever comes first. KUH is the only neurosurgical care provider for its catchment population (approximately 890,000 inhabitants), and all patients experiencing symptoms warranting neurosurgical attention are referred to our clinic.

### Meta-analysis of the literature

In order to compare our experience and results with those reported by others, we performed a systematic search in the Pubmed-database for surgical series and case reports which assessed bAVM recurrence by using the search terms “(AVM OR arteriovenous malformation) AND surgery,” “de novo AND AVM,” “acquired and AVM,” and “AVM AND brain AND case.” All studies which presented the bAVM rate of recurrence in surgically treated patients with occlusion confirmed by digital subtraction angiography (DSA) were included and classified according to the age distribution of their patient population. Case reports of AVM recurrences after surgical excision were also recorded. Surgical series with five or less patients were ignored. Bibliographies were cross-referenced to make sure that all suitable studies were included. A forest plot was created using the OpenMeta[Analyst] software (http://www.cebm.brown.edu/openmeta/). Outcomes across studies were pooled using a random-effects model and reported with a 95% confidence interval (CI). Heterogeneity was assessed with Cochran’s *Q* test and *I*^2^ statistic.

### Statistics

For continuous variables, median and range were calculated and Mann–Whitney U-test was used for comparison. For categorical variables, proportions and percentages were calculated and chi-square test was used for statistical comparison. Statistics were calculated with SPSS 22.0 (IBM, Armonk, NY) software.

## Results

### KUH patient cohort

For this study, 146 patients with 147 surgically treated AVMs in 1981–2017 were reviewed. Out of these 147 AVMs, 52 (35.3%) were also treated with preoperative endovascular embolization. Three (2.0%) patients were treated with radiosurgery after surgical resection failed to remove the entire lesion. Surgical occlusion was achieved in 135/147 (91.8%) of AVMs, and these patients formed the basis of the study. Median age of the study population was 37 years (min 0, max 70), and 51/135 (37.8%) of the patients were female. Seventeen (12.6%) of the 135 bAVM patients were considered pediatric (18 years old or younger) at the time of the operation. These 135 patients were clinically followed up for a median of 1.27years (min 0.02, max 30.40, interquartile range 0.37–5.39). Fifty-six patients had imaging follow-up spanning longer than 3 months with a median imaging follow-up of 3.39 years (min 0.25, max 28.3, IQR 0.82–11.99). In the pediatric subgroup, the median clinical follow-up was 3.67 years (min 0.02, max 18.7, IQR 1.12–7.03). For the 9 pediatric patients with imaging follow-up spanning longer than 9 months, median imaging follow-up was 5.51 years (min 1.47, max 24.97, IQR 2.46–7.04). Median clinical follow-up for non-pediatric patients was 1.24 years (min 0.04, max 30.40, IQR 0.37–4.58). For the 47 non-pediatric patients with imaging follow-up spanning longer than 3 months, median imaging follow-up was 2.67 years (min 0.25, max 30.40, IQR 1.06–14.98). Median follow-up from patient records was 18.56 years (min 0.02, max 39.33, IQR 7.98–28.74). Patients treated after 2018 were not included in the study due to lack of sufficient possible follow-up (less than 5 years at the time of writing). The demographics and clinical presentation of the studied patients with complete surgical removal of the lesion are presented in Table 1.

**Table 1:** Demographics and the clinical presentation of surgically treated bAVM patients in KUH

Clinical variable	Recurrent AVM	No recurrence	*p*-value
Median age (range)	14.5 (3–28)	37.5 (0–70)	0.001
Sex (% of females)	50% (3/6)	37.5% (48/128)	0.415
Presentation with hemorrhage	100% (6/6)	57.0% (73/128)	0.039
Focal neurological deficit	33.3% (2/6)	36.5% (47/128)	0.617
Motor deficit	33.3% (2/6)	18.8% (24/128)	0.330
Sensory deficit	0% (0/6)	10.2% (13/128)	0.535
Visual field deficit	0% (0/6)	6.3% (8/128)	0.686
Cognitive impairment	0% (0/6)	5.5% (7/128)	0.720
Mean Spetzler-Martin grade	2.17	2.26	0.643

### Rate of bAVM recurrence

Six out of 135 (4.4%) patients with DSA-confirmed complete removal of the lesion were later diagnosed with a recurrent lesion for a rate of one recurrence in 89.55 patient-years of clinical follow-up or one recurrence in 64.07 patient-years of imaging follow-up. Out of the 17 pediatric (18 years or younger) patients in our study, 5 (29.4%) later developed a recurrent bAVM for a rate of one recurrence in 19.16 years of clinical follow-up or one recurrence in 12.58 years of imaging follow-up. For non-pediatric patients, 1 (0.85%) out of 118 patients later developed a recurrence for a rate of one recurrence in 441.5 years of clinical follow-up or 321.52 years of imaging follow-up. The mean delay from initial surgical removal of the bAVM to the diagnosis of a recurrent lesion was 7.46 years (89.5 months, range 23.8–188.3 months). In our series, no patient was intended to be treated solely with embolization.

### Clinical presentation at recurrence

Two out of 6 recurrent bAVMs were found with follow-up imaging, while one was diagnosed after the patient reported increasing numbness of the left leg. The other 3 presented with intracranial hemorrhage years after the initial surgical removal. One out of 3 hemorrhages from a recurrent bAVM led to a permanent neurological deficit which impairs the patient’s ability to work. Four patients had their recurrent lesions surgically resected, while one patient was treated with radiosurgery and another with combined endovascular embolization and radiosurgery. Occlusion was achieved with all recurrent lesions. Further detail on these patients with recurrent bAVMs is provided in Table 2.

**Table 2:** Recurrent bAVM-patients in the KUH cohort

Case	Age at first treatment	Initial presentation	Spetzler–Ponce grade	Initial treatment	Time from occlusion to diagnosis of residive lesion	Recurrent presentation	Recurrent treatment	Final occlusion
1^a^	17	Visual field loss, SAH	B	Surgery	23.6 months	ICH	Surgery	Yes
2	28	Hemiparesis, ICH	B	Surgery	182.2 months	ICH	Surgery	Yes
3	16	Hemiparesis, ICH	B	Surgery	80.2 months	Increasing numbness on the left side of the body	Surgery	Yes
4	14	Reduced level of consciousness, IVH	A	Surgery	91.1 months	ICH	Surgery	Yes
5	13	Headache, ICH	A	Surgery	79.9 months	Follow-up imaging	Endovascularembolization + radiosurgery	Yes
6	3	Headache, ICH	A	Surgery	65.7 months	Follow-up imaging	Radiosurgery	Yes

a. Patient suffered a recurrent hemorrhage abroad and subsequent surgery was performed by a local institution

### Factors associated with bAVM recurrence

**Fig. 1:**
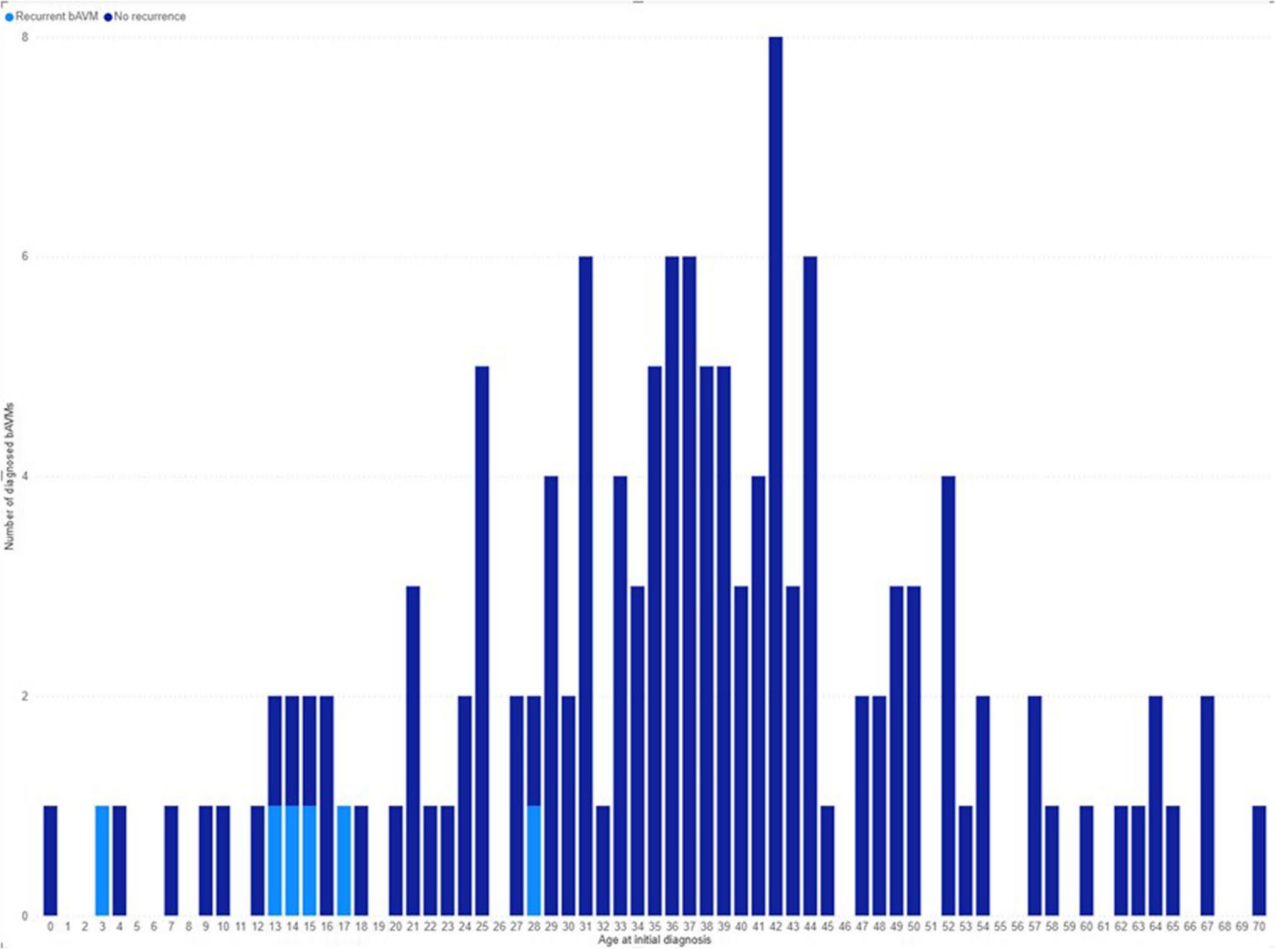
The age distribution of bAVM patients with complete surgical occlusion of their lesion upon initial diagnosis. The dark and light pillars demonstrate the incidence of bAVMs which were surgically excised in KUH in relation to patient age at initial diagnosis. Only bAVMs with complete, angiographically confirmed excision of the lesion are included. BAVM patients who later experienced a recurrence of their lesion are marked with a lighter color. This figure demonstrates that bAVMs carry a significant risk of recurrence after surgical excision in young patients

**Fig. 2:**
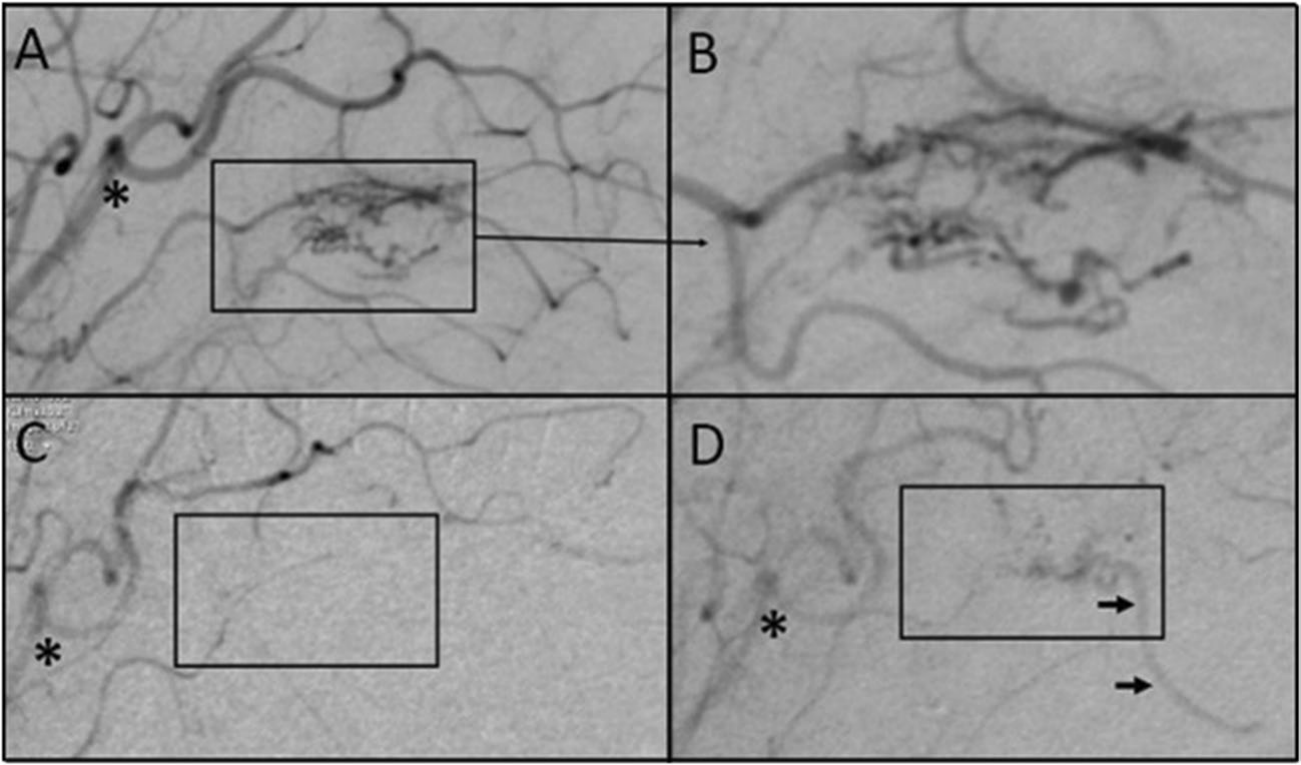
Example of bAVM recurrence in angiography. The preoperative digital subtraction angiography (DSA) of the bAVM is shown in **A** and **B**, with the latter being a magnified close-up of the area defined with the rectangle in A. Postoperative DSA without bAVM is shown in **C**, while **D** demonstrates the recurrent bAVM in a routine control DSA performed 5.5 years later. In order to make it easier to orientate to the anatomy, * marks the same arterial bifurcation in A, C, and D. The small black arrows in D point to the draining vein of the recurred bAVM, confirming the recurrence of a true arteriovenous shunt

Patients with later bAVM recurrence were significantly younger at first treatment than those without recurrence (median 14.5 years vs 37.5 years, *p* = 0.001), and they tended to present more often with intracranial or subarachnoid hemorrhage (100% vs 57.0%, *p* = 0.039). Half of the (3/6) bAVMs which recurred drained into the deep venous system at the first clinical presentation before surgery. For 5/6 patients, the presence of venous deep drainage after recurrence did not change from the bAVM’s initial presentation, while for one patient this could not be assessed due to the patient living abroad during the diagnosis of a recurrence and thus the imaging being unavailable. Further demographic comparison between patients experiencing a recurrent lesion and the rest of the study population is provided in Table 1. A visualization of the age distribution of bAVM patients in our institution and the prevalence of recurrent lesions within different age groups is provided in Fig. 1. An example of a bAVM recurrence in angiography is provided in Fig. 2.

### Systematic review of the literature on bAVM recurrence

**Fig. 3:**
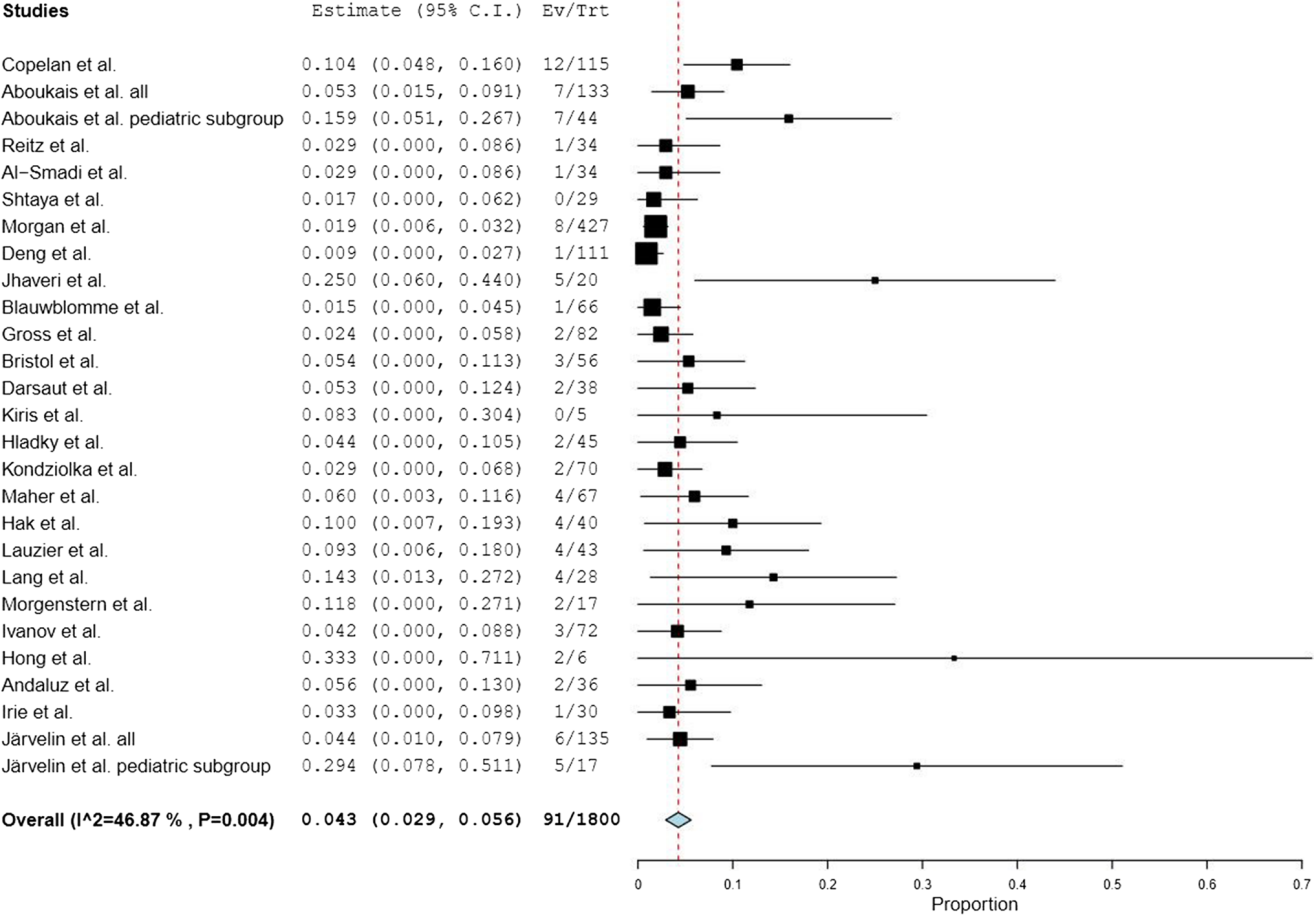
A forest plot demonstrating the rate of bAVM recurrence in the meta-analysis. The studies included in the meta-analysis are described on the left; 95% confidence intervals for each study as determined by the random effects model are provided. Ev/Trt, events/treatments

Twenty-five studies found in the literature and our own patient cohort were combined to present 26 surgical studies with, altogether, 1739 surgically treated bAVMs with DSA-confirmed occlusion. Seventy-nine out of 1739 patients later developed a recurrent bAVM, leading to an overall rate of recurrence of 4.5%. When considering only the pediatric subgroup of the review with one another study including patients younger than 25 years old included, 937 patients with 65 recurrent lesions were found, leading to a pediatric rate of recurrence of 6.9%. If only studies with patients of all ages were included in the analysis, 26 out of 863 bAVMs recurred for a rate of recurrence of 3.0%. Within the surgical studies, the mean delay from DSA-confirmed obliteration to diagnosis of the recurrent bAVM was 36.9 months (3.1 years). Among those surgical series in which suitable information could be gathered, 44 out of 50 (88%) recurrent bAVMs had their primary lesion initially present with a hemorrhage. Further information on these studies is presented in Table 3. A forest plot demonstrating the rate of recurrence in these studies is provided in Fig. 3.

**Table 3:** Surgical series included in the literature analysis

Author	Age group	Patients	Patients with recurrences	Mean time from treatment to recurrence (months)	Prevalence of initial hemorrhagic presentation among recurrences
Copelan et al. [6]	Under 25	115	12 (10.4%)	54,7	12 (100%)
Aboukaïs et al. [7]	All ages	133	7 (5.3%)	18,8	7 (100%)
Aboukaïs et al.^a^ [7]	Pediatric	44	7 (15.9%)	18,8	7 (100%)
Reitz et al. [9]	Pediatric	34	1 (2.9%)	3	N/A
Al-Smadi et al. [10]	Pediatric	34	1 (2.9%)	12	1 (100%)
Shtaya et al. [11]	Pediatric	29	0 (0%)	N/A	N/A
Morgan et al. [12]	All ages	427	8 (1.9%)	41,4	6 (75%)
Deng et al. [13]	Pediatric	111	1 (0.9%)	9	N/A
Jhaveri et al. [14]	Pediatric	20	5 (25%)	69,7	N/A
Blauwblomme et al. [15]	All ages	66	1 (1.5%)	N/A	N/A
Gross et al. [16]	Pediatric	82	2 (2.4%)	N/A	N/A
Bristol et al. [17]	Pediatric	56	3 (5.4%)	40	N/A
Darsaut et al. [18]	Pediatric	38	2 (5.3%)	N/A	N/A
Kiris et al. [19]	Pediatric	5	0 (0%)	N/A	N/A
Hladky et al. [20]	Pediatric	45	2 (4.4%)	N/A	N/A
Kondziolka et al. [21]	Pediatric	70	2 (2.9%)	36	N/A
Maher et al. [22]	Pediatric	67	4 (6.0%)	48	N/A
Hak et al. [23]	Pediatric	40	4 (10%)	N/A	N/A
Lauzier et al. [8]	Pediatric	43	4 (9.3%)	N/A	4 (100%)
Lang et al. [24]	Pediatric	28	4 (14.3%)	4,52	2 (50%)
Morgenstern et al. [25]	Pediatric	17	2 (11.8%)	45	1 (50%)
Ivanov et al. [26]	All ages	72	3 (41.7%)	28	3 (100%)
Hong et al. [27]	Pediatric	6	2 (33.3%)	54	N/A
Andaluz et al. [28]	Pediatric	36	2 (5.6%)	48	2 (100%)
Irie et al. [29]	All ages	30	1 (3.3%)	120	0 (0%)
Järvelin et al.	All ages	135	6 (4.4%)	89,5	6 (100%)
Järvelin et al.^a^	Pediatric	17	5 (29.4%)	68,1	5 (100%)

a. Pediatric subgroup within the study

## Discussion

We studied the rate of bAVM recurrence after angiographically confirmed, seemingly complete surgical resection. Our results show that bAVM recurrence is a relatively common phenomenon in pediatric patients.

### Cause of bAVM recurrence?

The mechanics behind the development of an AVM or a recurrence are still unknown, although several theories have been created in an attempt to explain the development of the lesion. Pellettieri at al. suggested that in a minority of AVMs, angiographically unfilled compartments exist alongside the angiographically visible lesion. Hemodynamic changes could induce blood flow into these compartments, filling the “hidden compartments” and resulting in the growth or recurrence of an AVM in angiography [30]. Others have suggested an angiogenic mechanism behind the development of AVMs. For example, Sonstein et al. demonstrated increased expression of vascular endothelial growth factor in histological samples from recurrent AVMs in comparison to non-recurrent AVMs [31]. The concept of aberrant angiogenesis as the cause of bAVM recurrence is supported by the landmark discovery by Nikolaev et al., who demonstrated the presence of somatic activating KRAS mutations in AVM patients and observed a connection between mutant KRAS expression and increased extracellular signal-regulated kinase activity leading to increased expression of genes related to angiogenesis in endothelial cells [32]. There are also several reports of “de novo” AVMs in the literature, suggesting that instead of being strictly congenital AVMs may also develop later in life [33]. This also supports the concept of aberrant angiogenetic growth as the source of bAVM recurrence, obviating the need for undiagnosed congenital bAVM compartments remaining after initial complete cure.

### Clinical implications

The overall hemorrhage risk of an unruptured, untreated AVM has been estimated to be 1–3% per year [34], with the risk being 2- to 2.5-fold in previously ruptured patients [35]. While data on the rupture risk and outcome of recurrent AVMs specifically is lacking, the outcomes in our cohort (1 out of 3 ruptures led to a deficit severe enough to affect the patient’s ability to work) and the general data on the outcomes of AVM hemorrhages create strong pressure toward vigorous follow-up imaging in a selective cohort of AVM patients. In a recent study by Karlsson et al., 20% of AVM-ruptures were fatal, with 45% of patients developing a new neurological deficit or experiencing worsening of a previous deficit. Only 1 out of 3 patients recovered completely [36].

Our review and own patient cohort support the notion of AVM recurrence being quite common in pediatric patients. The duration and frequency of imaging follow-up vary greatly in the literature, and the exact prevalence of recurrence varies between 0 and 33%. It is likely that the risk of recurrence has, thus far, been underestimated and that more vigorous follow-up schemes would result in a higher observed rate of recurrence. In our own patient cohort with a defined, relatively homogenic population, the delay from AVM occlusion to diagnosis of recurrence was quite high, yet prevalence of recurrence was also higher than generally reported in the literature. It is possible that other population-based factors may also factor into the prevalence of recurrence. While our patient cohort agrees with current literature on the increased prevalence of hemorrhage at initial bAVM presentation in recurrent lesions, unlike Morgan et al. [12], we did not find deep venous drainage more common in recurrent bAVM patients.

While recurrent AVMs, as reported in the literature, are far more likely to develop in pediatric patients, they may also present themselves in older patients. Our study demonstrates an AVM patient who was 28 years old at initial surgery and 43 years old during the diagnosis of a recurrence. While quite old in comparison to the general population of recurrent AVMs, he is not the oldest as Morgan et al. [12] report a patient who had her initial AVM excised at 42 years old and experienced a recurrent lesion 33 months later.

These older patients create a dilemma: what are the criteria with which we should subject our surgically treated AVM patients to follow-up imaging, and how should one go about designing that follow-up? In addition to the golden standard, digital subtraction angiography (DSA), magnetic resonance imaging (MRI) has been used to follow-up on AVM patients. While the most accurate method of imaging when it comes to AVMs, DSA is an invasive procedure which exposes patients to ionizing radiation and procedural complications with approximately 2.6% experiencing a neurological event within 24 h of the procedure [37]. The procedure involves a 0.14% chance of permanent stroke and a 0.05% likelihood of death related to a neurological condition [37]. It is also more time consuming and expensive to perform. While anesthesia may be required in children to obtain proper imaging, magnetic resonance imaging is noninvasive.

The main issue with MRI is its accuracy, which is lacking in comparison to DSA; 4D MRA performed at 3T has been demonstrated to have a sensitivity of 73.7% and specificity of 100% in comparison to DSA in detecting residual lesions after radiosurgery [38], while in another study TOF-MRA had a sensitivity of 50% and specificity of 96.1% and contrast-enhanced MRI a sensitivity of 84.6% and specificity of 38.5% in comparison to DSA in diagnosing a recurrent AVM [14]. Contrast-enhanced MRI is more specific, but there have been concerns about emerging evidence of gadolinium deposition after repeated contrast-enhanced imaging [39]. While MRI is noninvasive and more accessible, it has been suggested that performing a delayed postoperative DSA a year after surgery may detect a significant portion of early recurrences which are too small to be detected with MRI [24].

### Limitations of this study

There is likely selection bias included in our patient cohort. Extensive imaging follow-up was not always carried out in our patient cohort, and median clinical follow-up in our patient cohort is relatively short. However, median follow-up from patient records was 18.56 years and given that our institution is the sole neurosurgical care provider for the population of eastern Finland, it is reasonable to assume that patients with a symptomatic bAVM recurrence have been referred to our clinic. The real rate of recurrence for bAVMS may be higher than what is presented in this study.

## Conclusions

BAVMs may recur after complete angiographic cure in a fairly high percentage of pediatric bAVM patients. Since the bAVM recurrence may lead to a debilitating hemorrhage, long-term follow-up of treated bAVMs, especially pediatric ones, is recommended even after complete angiographic cure.
